# The effect of communicating the genetic risk of cardiometabolic disorders on motivation and actual engagement in preventative lifestyle modification and clinical outcome: a systematic review and meta-analysis of randomised controlled trials

**DOI:** 10.1017/S0007114516002488

**Published:** 2016-07-13

**Authors:** Sherly X. Li, Zheng Ye, Kevin Whelan, Helen Truby

**Affiliations:** 1 Medical Research Council Epidemiology Unit, University of Cambridge, Cambridge CB2 0QQ, UK; 2 Diabetes and Nutritional Sciences Division, King’s College London, London SE1 9NH, UK; 3 Department of Nutrition & Dietetics, Monash University, Level 1, 264 Ferntree Gully Road, Notting Hill, VIC 3168, Australia

**Keywords:** Genetic risk, Behaviour change, Systematic reviews, Cardiometabolic disorders

## Abstract

Genetic risk prediction of chronic conditions including obesity, diabetes and CVD currently has limited predictive power but its potential to engage healthy behaviour change has been of immense research interest. We aimed to understand whether the latter is indeed true by conducting a systematic review and meta-analysis investigating whether genetic risk communication affects motivation and actual behaviour change towards preventative lifestyle modification. We included all randomised controlled trials (RCT) since 2003 investigating the impact of genetic risk communication on health behaviour to prevent cardiometabolic disease, without restrictions on age, duration of intervention or language. We conducted random-effects meta-analyses for perceived motivation for behaviour change and clinical changes (weight loss) and a narrative analysis for other outcomes. Within the thirteen studies reviewed, five were vignette studies (hypothetical RCT) and seven were clinical RCT. There was no consistent effect of genetic risk on actual motivation for weight loss, perceived motivation for dietary change (control *v.* genetic risk group standardised mean difference (smd) −0·15; 95 % CI −1·03, 0·73, *P*=0·74) or actual change in dietary behaviour. Similar results were observed for actual weight loss (control *v.* high genetic risk SMD 0·29 kg; 95 % CI −0·74, 1·31, *P*=0·58). This review found no clear or consistent evidence that genetic risk communication alone either raises motivation or translates into actual change in dietary intake or physical activity to reduce the risk of cardiometabolic disorders in adults. Of thirteen studies, eight were at high or unclear risk of bias. Additional larger-scale, high-quality clinical RCT are warranted.

Personalised nutrition has been described as nutritional advice formulated according to an individual’s characteristics or that of a population subgroup^(^ ^1^ ^)^. Such personal characteristics may include phenotypic features and dietary preferences, with age and sex as features of personalisation evident in current nutritional guidelines^(^ ^1^ ^)^. Recently, genetics has been proposed to help further refine personalised nutrition. High expectations have been expressed regarding the potential for translating research about lifestyle–gene interactions into personalised nutrition and also that learning about personalised genetic risk may increase the adoption of healthy lifestyle behaviours^(^ ^2^ ^–^ ^5^ ^)^. Certainly, genetic risk may be a potent motivator for behaviour change because of its biological accuracy and personal salience, which is consistent with the Health Belief Model^(^ ^6^ ^)^. On the basis of this, a burgeoning number of companies are providing direct-to-consumer (DTC) genetic testing, which appear to be gaining popularity with the public, despite the clinical validity and utility being as yet unclear^(^ ^7^ ^,^ ^8^ ^)^.

Earlier studies have indicated that the provision of personalised genetic information favourably influences screening behaviours and medication adherence for individuals at risk of familial cancers, often involving Mendelian inheritance with high-penetrance genetic variants^(^ ^9^ ^)^. However, this cannot be assumed for the adoption of more complex ‘lifestyle’ health-related behaviours, such as dietary modification, which are required to be adopted and sustained in order to reduce the risk of developing cardiometabolic disorders such as obesity, type 2 diabetes (T2D) and CVD. These highly prevalent conditions have low-penetrance susceptibility genetic variants plus a multifactorial aetiology. A cochrane systematic review in 2010 found little or no effect of the provision of genetic disease risk estimates on change in physical activity or dietary behaviours, although from a limited number of available studies with poor quality^(^ ^10^ ^)^. This and other reviews make similar conclusions, including the largest DTC-based cohort study to date (*n* 2037)^(^ ^11^ ^–^ ^14^ ^)^. Although these were largely based on vignette studies (where participants were provided with an imaginary scenario of their genetic risk), recently the evidence has been enhanced by a number of clinical intervention studies.

At present, many DTC companies are providing genetic testing for multifactorial conditions predicated on the above hypothesis in order to meet public demand^(^ ^15^ ^)^, but dangerously they are embedded in an environment without regulatory frameworks to protect against misuse of DTC services^(^ ^16^ ^)^. Therefore, a systematic evaluation that captures these newer studies is warranted to help clarify the motivational impact of genetic risk information and its effect on actual behaviour and clinical outcome.

Therefore, we performed a systematic review and meta-analysis of randomised controlled trials (RCT) undertaken in the context of cardiometabolic disorders (obesity, T2D, CVD) to investigate the following: (1) the effect of genetic risk testing and communication on perceived and actual motivation to engage in risk-reduction lifestyle modification (diet and physical activity); and (2) the effect of genetic risk testing and communication on actual lifestyle modification and clinical outcomes.

## Methods

The protocol of this systematic review and meta-analysis was registered with the University of York, Centre for Reviews and Dissemination PROSPERO database (CRD42014009096)^(^ ^17^ ^)^.

### Eligibility

#### Types of participants

Given the limited knowledge of the impact of genetic risk communication on multifactorial conditions with reduced-penetrance susceptibility genetic variants such as obesity, T2D and/or CVD, we were interested in examining any individual, either healthy or at risk of these disorders, in the context of disease prevention. No restrictions were placed on age, sex or ethnicity.

#### Types of interventions and comparators

We deemed any RCT assessing the provision of genetic risk prediction information for the aforementioned disorders as eligible. Studies could either involve a clinical genetic test, where participants undertook a real genetic test and were provided with their actual results or a vignette, which was defined as a hypothetical scenario providing a fictitious but plausible genetic risk.

The intervention (genetic risk information) could be compared with either a control (no genetic risk information) or alternative risk information (e.g. hormone or enzyme) or both.

#### Outcome measures

These included motivation for, or actual, lifestyle behaviour change (diet, physical activity or health screening) and any physiological or clinical outcome that would result from this lifestyle behaviour change (e.g. change in body weight, HbA1c or blood pressure).

As only RCT, representing the highest level of primary evidence were included, studies of any other design, those relating to new-born screening, family history analysis or investigating the efficacy of diet–gene interactions were excluded. Addictive behaviours (e.g. smoking) and non-lifestyle-related behaviours (e.g. medication adherence) were excluded.

### Identification of studies

All RCT published on this topic, between 2003 (following completion of the Human Genome Project) and June 2015, were identified without the limitation of language, length of intervention and/or follow-up. Electronic searches using MEDLINE, PsycINFO, EMBASE, CINAHL+, Cochrane Central Register of Controlled Trials combined search terms related to genetic risk, health behaviour and either obesity, T2D and/or CVD (see the online Supplementary material 1 for a sample search strategy). Inclusion of both keywords and medical subject headings ensured a comprehensive search. The grey literature was searched using the key terms applied to MEDLINE, including ProQuest, Trove, ETHOS and Science.gov. Reference lists from previous reviews were also mined for eligible studies. Prominent authors identified from subject knowledge and relevant reviews were searched by name. Unpublished studies were identified via the WHO International Clinical Trials Registry platform, and the authors of completed but so far unpublished studies were contacted for more information (via email and a reminder was sent if there was no response after 2 weeks) (four contacted/one responded). In addition, we contacted authors of published studies that had incomplete data for our meta-analyses; thus, we attempted to minimise publication bias (five contacted/four responded).

### Study selection

Studies were screened by title and abstract by two independent reviewers, against the eligibility criteria, and if selected by both reviewers the full-text was reviewed. Any disagreements regarding eligibility were resolved by discussion. All studies were eligible for meta-analysis, but meta-analysis was only undertaken if there were at least two studies assessing the same outcome.

### Data collection

Data extraction for each study followed a standard procedure, where two reviewers independently extracted data according to a specific proforma (Table 1). Any discrepancies between the reviewers were resolved by discussion, with the involvement of two other reviewers when consensus could not be reached. If investigated outcomes (as per protocol) were not reported within their publication, authors were contacted to request for further information.

**Table 1:** Data extraction items

Items in the proforma	Data items extracted
Background	Source of funding, study design, aim
Method	Location, number of participants, population characteristics, length of follow-up, source of genotyping/genetic risk counselling, dietary assessment method, genetic assessment method, targeted disease, measurement of outcome, intervention (type of genetic or alternative risk information) and comparator
Risk of bias	Sequence generation for randomisation, allocation concealment, effective blinding, completeness of outcome data, free from selective reporting, other biases
Results	Mean, standard deviation, CI, *P*-value, adjustment for confounders
Conclusion	Author conclusions and reviewers’ interpretation

### Risk of bias assessment

Studies were assessed using the Cochrane Collaboration tool for assessing risk of bias in RCT^(^ ^18^ ^)^. A summary statement indicating the sources of bias for each study was created according to the key areas of bias advised (Table 2); two authors independently assessed bias at the study level. This information was used to interpret findings as well as indications for sensitivity analysis.

**Table 2:** Summary of risk of bias judgements for studies included in this review

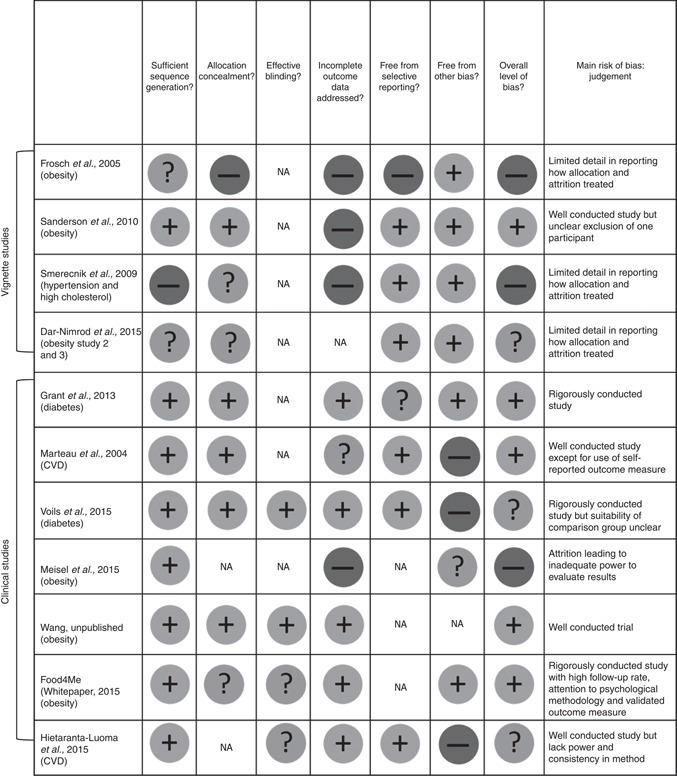

NA, not available; 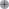, low risk of bias; 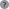, unclear risk of bias; 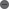, high risk of bias.

### Measuring the effect of intervention and method of analyses

Given that anticipated behaviours from hypothetical scenarios do not necessarily result in actual behaviour change and this is a known limitation of vignette studies^(^ ^19^ ^)^, results from vignette and clinical RCT are analysed and presented separately. Therefore, we have distinguished between perceived *v*. actual motivation for change to more accurately reflect the interpretation of vignette and clinical studies, respectively. A primarily narrative approach to analysis would be undertaken, as pre-specified in the protocol, if high heterogeneity existed between studies. A meta-analysis was conducted after homogenising comparable data (Stata Statistical Software, version 13; StataCorp LP). The principal summary measure was standardised mean difference (smd), centred on 0, with values >0 favouring the intervention group (genetic risk communication) and values <0 favouring the comparison group. To take into account heterogeneity across studies, a random effects meta-analysis was used by combining results for studies of varying interventions^(^ ^20^ ^)^. All the analyses were conducted using the available case analysis^(^ ^20^ ^)^. Where results were measured at multiple time points, the furthest point in time was used in the meta-analysis to represent effects on long-term behaviour change. Heterogeneity was evaluated using *χ* ^2^ tests and *I* ^2^ statistic. If high levels of heterogeneity existed, possible sources of heterogeneity were identified (e.g. high risk of bias, comparability of control group, method of risk communication) followed by narrative rather than statistical sensitivity analysis. Where possible, we have stratified analysis according to the level of genetic risk, because genotype had been previously identified as a potential effect modifier^(^ ^10^ ^)^. Formal statistical publication bias was not undertaken because of insufficient number of studies per outcome of interest.

## Results

### Study selection

**Fig. 1:**
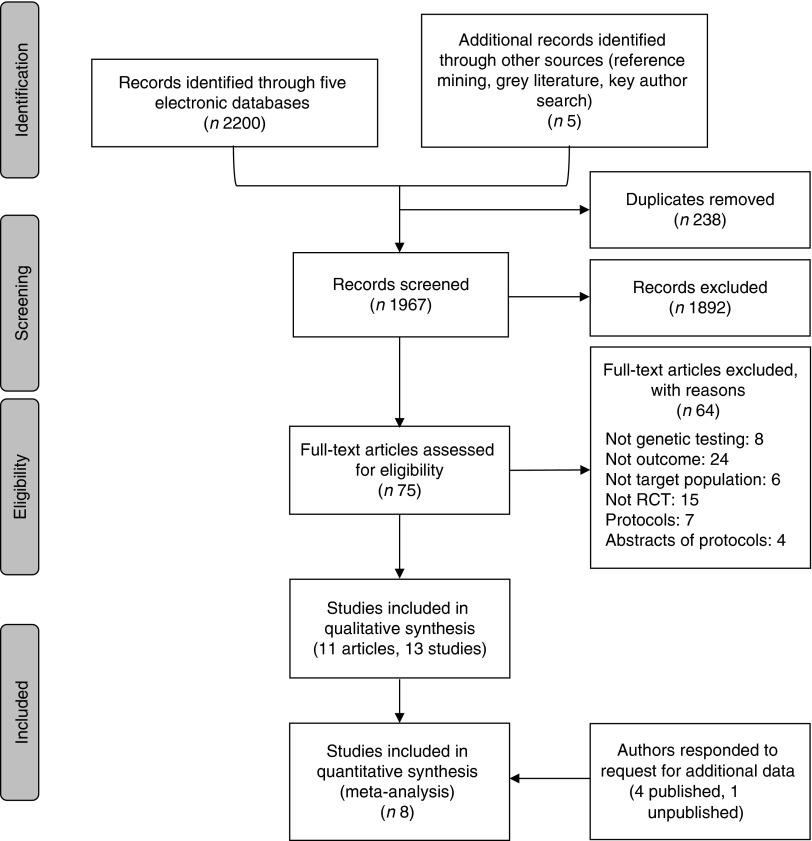
Flow chart of studies identified and included in the systematic review and meta-analysis. RCT, randomised controlled trials.

In total, 1967 unique citations were screened for inclusion, and eleven publications, representing thirteen unique studies, form this review (see Fig. 1 and Table 3).

**Table 3:** Summary of studies reporting on genetic risk communication and lifestyle behaviour change

Outcomes	Clinical studies*	Vignette studies*	No. of participants†	Average age (years)	Ethnicities reported
Perceived motivation to change behaviour					
Obesity	0	Sanderson^(^ ^28^ ^)^, Frosch^(^ ^27^ ^)^	440	24·9	White, Asian, African American, others
T2D	0	0	0	–	
CVD	0	Smerecnik^(^ ^30^ ^)^×3	432	33·2	
Total number	0	5	872		
Actual motivation to change behaviour					
Obesity	Wang^(^ ^21^ ^)^, Meisel^(^ ^29^ ^)^	NA	975	35·5	African American, White, others
T2D	Grant^(^ ^25^ ^)^	NA	108	58·7	
CVD	0	NA	0	–	
Total number	3	NA	1083		
Risk reducing behaviour (dietary, physical activity or other)					
Obesity	Celis-Morales^(^ ^22^ ^)^, Meisel^(^ ^29^ ^)^	Dar-Nimrod^(^ ^32^ ^)^	2048	27·2	African American, White, Asian, others
T2D	Voils^(^ ^23^ ^)^, Grant^(^ ^25^ ^)^	0	709	56·4	
CVD	Marteau^(^ ^26^ ^)^, Hietaranta-Luoma^(^ ^24^ ^)^	0	423	51·0	
Total number	6	1	3180	–	
Clinical outcome (BMI, weight loss, HbA1c)					
Obesity	Wang^(^ ^21^ ^)^, Meisel^(^ ^29^ ^)^, Celis-Morales^(^ ^22^ ^)^	0	2582	36·9	African American, White, others
T2D	Voils^(^ ^23^ ^)^, Grant^(^ ^25^ ^)^	0	709	56·4	
CVD	0	0	0	–	
Total number	5	0	3291		
Genetic loci examined (either genotyped or used as within a hypothetical scenario)	*FTO, TCF7L2, PPARγ, KCNJ1, ApoE, LDAR, ApoB, GATA-2, FTO, KLF15* and a few fictitious genes				
Total overall	14	6	8426	42·2	

T2D, type 2 diabetes mellitus; NA, not applicable; *FTO*, fat mass and obesity associated gene; *TCF7L2*, transcription factor 7-like 2; *KCNJ11*, potassium channel, inwardly rectifying subfamily J, member 11; *LDAR*, LDL receptor; *GATA-2*, GATA binding protein 2; *KLF15*, Kruppel-like factor 15.

* The last name of each study’s first author is listed.

† Number of participants based on available case analysis.

### Characteristics of included studies

Of the thirteen included trials, six were vignette studies (hypothetical scenarios) and seven were clinical intervention studies. This included one unpublished^(^ ^21^ ^)^ and one semi-published study^(^ ^22^ ^)^ where authors agreed to contribute data. The detailed characteristics of included trials are shown in Table 3 and in the online Supplementary materials 2 and 3.

All participants were recruited from the general population, where the majority of participants in vignette studies were university students and in the clinical studies were middle aged; four of the seven clinical studies recruited a ‘high-risk population’ (e.g. overweight or met a criterion for having the metabolic syndrome) but who did not have the condition of interest at baseline^(^ ^23^ ^–^ ^26^ ^)^. The average length of follow-up for clinical studies was 6 months. On the basis of available case analysis, the cumulative sample size for all studies was 8426, ranging from 107^(^ ^24^ ^)^ to 1607^(^ ^22^ ^)^ (median 249).

Participants were either randomised to two groups, comparing those provided with genetic test results and a control group (either phenotypic risk feedback, standard healthy lifestyle advice or no risk feedback), or three or more groups comparing the aforementioned with feedback from an alternative test; two of the vignette^(^ ^27^ ^,^ ^28^ ^)^ and four of the clinical studies^(^ ^21^ ^,^ ^24^ ^,^ ^25^ ^,^ ^29^ ^)^ presented results stratified by the level of genetic-conferred risk (online Supplementary materials 2 and 3). For example, participants could possess 0, 1 or 2 risk alleles for a SNP associated with the condition (e.g. *fat mass and obesity associated* gene for obesity risk), or were categorised according to a composite genetic risk score. Genetic risk was assigned via a hypothetical scenario within vignette studies and by genotyping within clinical studies. All outcomes of interest were examined in studies investigating obesity and T2D, whereas four studies assessed perceived motivation and actual behaviour change in the context of CVD. A range of real and fictitious genetic variants was used.

### Risk of bias

There were similar numbers of studies with low (*n* 5), high (*n* 4) and unclear risk of bias (*n* 4) (Table 2). Vignette studies were more prone to bias (three high risk, one low risk) than clinical studies (one high risk, four low risk). Attrition bias was prevalent due to loss to follow-up and/or inadequate explanation for excluding certain participants. In all, five clinical studies had published protocols.

### Trial outcomes

For each of the outcomes examined, results from the clinical studies will be followed by results from the vignette studies.

#### Motivation to change behaviour

##### Clinical studies

In clinical studies, ‘actual motivation’ was assessed after participants undertook genetic testing and were provided personalised genetic results. In the clinical studies, weight-loss motivations were mixed. In two of the studies reviewed, participants who were provided genetic risk feedback were reported to possess higher motivation to lose weight (Wang *et al*., unpublished result) or stage of change for weight control (OR 1·77; 95 % CI 1·08, 2·89, *P*=0·023) compared with controls^(^ ^21^ ^,^ ^29^ ^)^. In that study, motivation was accentuated in those with a genotype for elevated risk (AA/AT *v.* control OR 2·38; 95 % CI 1·33, 4·26, *P*=0·003)^(^ ^29^ ^)^. This result was not evident in another study where diabetes prevention was the focus^(^ ^25^ ^)^. Similarly, motivational intent for improving diet and exercise appeared unaffected by genetic risk information across relevant studies^(^ ^25^ ^)^. These studies were not meta-analysed because of insufficient number of studies with comparable outcome measures.

##### Vignette studies

**Fig. 2:**
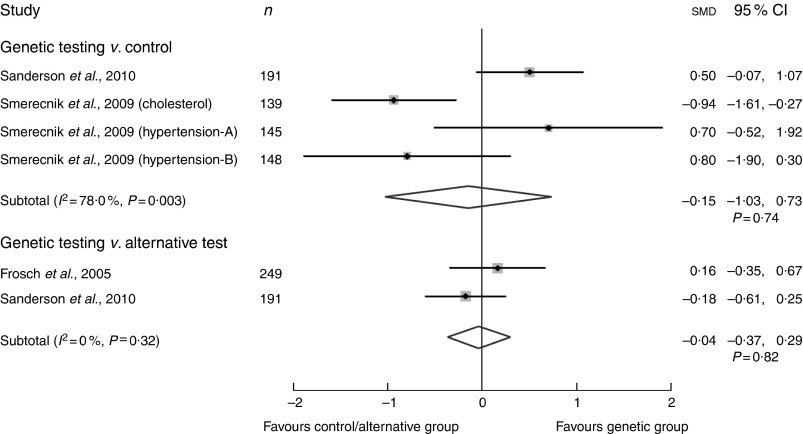
Summary of pooled standardised mean difference (SMD) in perceived motivation to change dietary behaviour via a random effects meta-analysis of vignette studies (standardised Likert scale: 1–10). *I* ^2^, between-trial heterogeneity.

In vignette studies, participants’ ‘perceived motivation’ was assessed after provision of a hypothetical genetic test or supposed factual information about the genetic aetiology of a disease. First, the effect of genetic risk information compared with controls (not receiving genetic information) on motivation for dietary modification was examined in four studies^(^ ^28^ ^,^ ^30^ ^)^. Although both groups reported a high motivation to change (>7 out of 10 on a Likert scale), our random effects meta-analysis showed that those with genetic risk information had a slight but non-significantly lower motivation to change compared with the control (smd −0·15; 95 % CI −1·03, 0·73, *P*=0·74) (Fig. 2 and online Supplementary material 2)^(^ ^28^ ^,^ ^30^ ^)^. High heterogeneity was evident, *I* ^2^=78 %, *P*=0·003, thereby reducing confidence in the pooled null finding. This may be due to a study with high risk of bias and adopting a non-personalised approach in communicating risk^(^ ^29^ ^)^. In addition, it may reflect age-related differences where participants of studies favouring genetic risk were younger (aged 20s) (Sanderson *et al*., and Smerecnick *et al*., study on hypertension A) compared with those favouring the control (aged 40s) (Smerecnik *et al*., study on cholesterol and hypertension B) who were older. Second, the difference in ‘perceived motivation’ for dietary modification after provision of risk from either a genetic test or an alternative test was also examined. Meta-analysis of two studies with conflicting findings showed a SMD of −0·04; 95 % CI −0·37, 0·29, *P*=0·82, with no indication of heterogeneity (*I* ^2^=0 %) (Fig. 2)^(^ ^27^ ^,^ ^28^ ^)^.

#### Actual behaviour change

##### Clinical studies

Among the six clinical studies with interventions ranging from 1 to 12 months, there were inconsistencies regarding whether learning about genetic risk alters dietary intake and/or physical activity (online Supplementary material 3). Among them, three studies reported differences in self-reported dietary intake between the genetic and control groups^(^ ^23^ ^,^ ^26^ ^,^ ^31^ ^)^ while two others did not^(^ ^22^ ^,^ ^24^ ^)^.

The results from a study among 601 veterans at risk of T2D reported a borderline statistically significant difference in macronutrient and total energy intakes at 3 months; however, this was not reported to be sustained at 6 months (difference in log energy −0·1; 95 % CI −0·1, 0, *P*=0·20)^(^ ^23^ ^)^. In another study of 316 probands (the first family member affected by a genetic disorder) who were diagnosed with familial hypercholesterolaemia (with considerably high genetic penetrance for CVD), the authors did not observe any difference in the proportion of participants that chose to follow a low-fat diet 6 months after genetic counselling^(^ ^26^ ^)^. On the other hand, results from a four-arm, web-based RCT (*n* 1269 healthy Europeans) reported that overall dietary quality (Healthy Eating Index: genetic group was 1·4 units higher than the control, *P*<0·01) and salt and fat intakes significantly improved in those who were provided genetic risk information compared with controls^(^ ^31^ ^)^. However, the authors reported negligible differences between all personalised nutrition groups at the end of the study (levels 1–3; online Supplementary material 3). Another Finnish study (*n* 107) concurred with such inconsistencies. In a subgroup analysis by genotype, they revealed that those possessing a high-risk genotype (E4+) reported consuming greater quantities of dietary fat compared with those with low-risk genotype (E4−) and similarly compared with the control group (*P*<0·05)^(^ ^24^ ^)^. Interestingly, no significant difference was found for quality of fat intake, vegetables and fruits, or alcohol intake^(^ ^24^ ^)^. Therefore, regardless of the limited number of studies reviewed, there is an inconsistent impact of genetic risk on dietary behaviour. Any benefit of which appears short term and only if compared with interventions lacking personalisation. The heterogeneity in dietary intake measurement did not enable meta-analysis for this outcome.

Of the five clinical studies^(^ ^24^ ^–^ ^26^ ^,^ ^29^ ^)^ measuring changes in physical activity after genetic risk communication, only one reported their findings, indicating no substantial effect on physical activity^(^ ^24^ ^)^. Only one study precluded meta-analysis.

##### Vignette studies

A vignette study subjected 162 Canadian undergraduate students to a psychological ‘cookie eating’ experiment^(^ ^32^ ^)^. Participants were randomised into three groups who received a newspaper article where obesity was described on the basis of either (i) its genetic or (ii) its psychosocial aetiology or (iii) a control where body weight was not mentioned. Despite the hypothetical nature of this experiment, the group that was influenced to consider obesity as genetically driven consumed significantly more cookies (mean 52·0 (sd 41·8) g) than the psychosocial group (mean 33·1 (sd 22·9) g, *P*=0·02) who were only marginally different to the control group (mean 37·0 (sd 29·8) g, *P*=0·08), after adjustment for sex, age and self-reported BMI^(^ ^32^ ^)^.

#### Clinical outcome

##### Clinical studies

**Fig. 3:**
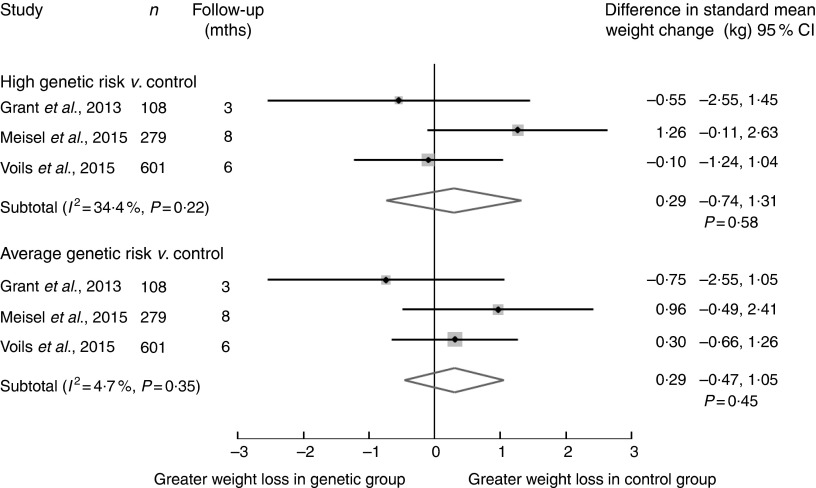
Summary of pooled standard mean difference in weight change between genetic *v.* control groups via a random effects meta-analysis of clinical studies (weight change in kg). *I* ^2^, between-trial heterogeneity; mths, months.

Weight loss was examined in five clinical studies, three of which investigated obesity prevention^(^ ^21^ ^,^ ^29^ ^)^. Preliminary results from the European study ‘Food4Me’ reported that there was no statistically significant difference in the 6-month weight change between intervention and control groups, including in those who were overweight and/or obese at baseline^(^ ^31^ ^)^. Our meta-analysis of three studies comparing those provided genetic risk (either high or average risk) with control groups demonstrated no difference (Fig. 3)^(^ ^23^ ^,^ ^25^ ^,^ ^29^ ^)^. There was a standard mean weight loss of 0·29 kg in favour of the control group, although with large uncertainty for those with high genetic risk compared with control, 95 % CI −0·74, 1·31, *P*=0·58, and minimal heterogeneity (*I* ^2^=34 %), and similar results were observed for those at average genetic risk compared with control.

Other clinical indicators including insulin resistance^(^ ^23^ ^)^ and attendance at a diabetes prevention programme^(^ ^25^ ^)^ did not differ between those provided genetic risk information and controls.

## Discussion

This review investigated the effect of communicating genetic risk on lifestyle modification for cardiometabolic disorders and found no evidence that this information improved participants’ dietary or exercise behaviour. It included recent, available clinical studies that build upon several related reviews, all of which concluded that there was limited support for such behavioural benefits^(^ ^10^ ^,^ ^13^ ^,^ ^33^ ^)^. Our findings are consistent with the updated Cochrane Systematic Review for dietary (smd 0·12; 95 % CI −0·00, 0·24, *P*=0·05) and physical activity behaviours (SMD −0·03; 95 % CI −0·14, 0·07, *P*=0·54) (published whilst our review was under consideration)^(^ ^34^ ^)^. Our review complements their results with two additional clinical RCT (Wang *et al*., unpublished results; Food4Me: *n* 1607)^(^ ^21^ ^,^ ^22^ ^)^, on top of five vignette studies. Moreover, given that all studies measured self-reported behaviours, which are subject to recall bias, our review extends beyond examining only behaviours to clinical outcomes (i.e. weight loss) that may indirectly reflect changes in lifestyle and non-lifestyle behaviours.

### Motivation for behaviour change

Findings for actual motivations regarding weight change/control were mixed, with two clinical studies showing that this was favourably influenced by genetic risk (Wang *et al*., unpublished results)^(^ ^29^ ^)^. The reason for this inconsistency may be explained by two potential mediators and/or moderators relating to participant characteristics: their initial level of motivation and their genetic literacy. First, qualitative evidence suggests that baseline motivational status may mediate motivational change, where individuals with low baseline motivation have comparatively less incentive to change lifestyle behaviour than those with higher baseline motivation^(^ ^35^ ^,^ ^36^ ^)^. A particular study demonstrated a significant benefit of genetic risk communication on weight-loss motivations only among those with underweight/normal BMI (Wang *et al*., unpublished results). This suggests that these participants may possess pre-existing motivations for a healthy lifestyle, which may indicate the possible transferable effect of motivation rather than an additive effect of genetic risk communication. Second, there is strong evidence that genetic literacy determines understanding of genetic risk and subsequent motivation to pursue healthy behaviours^(^ ^37^ ^,^ ^38^ ^)^. Vassy *et al*.^(^ ^38^ ^)^ noted that motivational response to low genetic risk results were dependent upon the participant’s genetic literacy, whereby those with low genetic literacy showed higher motivation for lifestyle modification. The two studies in our review that identified a motivational benefit from genetic risk communication were provided as an online risk feedback^(^ ^21^ ^,^ ^29^ ^)^, whereas the study that did not replicate this finding employed a qualified genetic counsellor to communicate risk^(^ ^25^ ^)^. As the probabilistic nature of genetic information is usually poorly understood, with only 38 % of US college-educated adults accurately interpreting their risk, and much lower when delivered online than in person^(^ ^39^ ^)^, this raises intriguing questions. DTC genetic services, which mostly use web-based delivery, have been criticised for their high literacy demands^(^ ^40^ ^)^ and are discouraged by several government organisations for providing genetic information without health professional support^(^ ^41^ ^)^. Questions remain as to how much of this difference can be attributed to the mode of delivery of risk information and whether online services alone can accommodate and support varying levels of genetic and health literacy.

### Actual behaviour change

There were mixed findings for dietary behaviour from the six clinical studies. Heterogeneity in type of dietary behaviour (e.g. percentage of energy intake, healthy eating index) precluded conducting a meaningful meta-analysis: three studies reported a benefit of genetic risk on adopting healthier eating behaviour. One study identified temporary borderline significant effects^(^ ^23^ ^)^. The second study suggested that the process of personalisation (i.e. tailoring advice) rather than the tool used to convey personalisation appears more important^(^ ^31^ ^)^. A third study highlighted modulation by level of genetic risk^(^ ^24^ ^)^. Indeed, within the studies reviewed, those that compared different forms of personalised risk information (i.e. genetic risk/counselling with an alternative risk/counselling)^(^ ^23^ ^,^ ^25^ ^)^ did not observe significant differences in dietary behaviour, unlike the studies that compared genetic risk/counselling with general health advice^(^ ^24^ ^,^ ^29^ ^)^. As personalising therapy is the basis of client-centred approaches, which have been found to better motivate change in various health behaviours and enhance outcomes at least in the short term^(^ ^42^ ^)^, this suggests that any form of personalisation may be beneficial in supporting behaviour change.

Several non-RCT studies also fail to identify any effect of genetic risk information on adopting healthy lifestyle behaviours, including a before-and-after study of 1325 employees from a DTC company who were provided genetic risk information but had no observed improvements in fat intake (*P*=0·34), exercise (*P*=0·39) or disease-screening behaviour (*P*=0·43) at 12 months of follow-up^(^ ^12^ ^,^ ^43^ ^)^. This finding concurs with evidence from other multifactorial conditions such as colorectal cancer^(^ ^44^ ^)^. This lack of behaviour change has led some to hypothesise that genetic risk itself may not be enough and that the provision of lifestyle advice based on genetics for how to mitigate this risk incurred by genetics would encourage adoption of the desired behaviours. For example, Zeevi *et al*.^(^ ^45^ ^)^, recently suggested that further personalisation of dietary advice using an algorithm derived from various personal factors such as sleep–wake cycle, physical activity and gut microbiota in addition to dietary habits can be used successfully to moderate glycaemic response in adults when delivered by a trained dietitian. This presents a new model for tailoring dietary advice that may be more robust than genetic risk alone. However, the utility of personalised nutrition, particularly using genetic data on behaviour change, is also unclear, with two small clinical trials funded by nutrigenetic companies, reporting improvements in self-reported dietary intake and BMI^(^ ^3^ ^,^ ^46^ ^)^. However, this was not replicated within a larger multinational personalised nutrition RCT (Food4Me), which adopted country-specific validated dietary assessment tools^(^ ^31^ ^)^. Hence, given that perceived risk (including that from genetic risk) does not strongly influence behaviour change^(^ ^47^ ^)^ and the limited ‘information value’ derived from the low predictive power of known genetic variants^(^ ^36^ ^,^ ^48^ ^,^ ^49^ ^)^, it may be unreasonable to expect genetic risk to have the profound impacts on behaviour that has been claimed.

Finally, a clinical study reported that elevated genetic risk resulted in higher consumption of dietary fat^(^ ^24^ ^)^ and another that informed participants of the genetic aetiology of disease led to increased unhealthy food consumption^(^ ^32^ ^)^. This may be explained by maladaptive coping in accordance with the ‘common sense model of illness cognition’, in which individuals’ belief about disease threats guides their prevention behaviour – that is, people can cope with disease threats broadly in two ways, either they reduce the threat by adapting to healthy behaviours or they form maladaptive mechanisms including fatalistic responses dependent on the perceived controllability of the threat^(^ ^50^ ^)^. However, recent meta-analyses and qualitative studies together indicate a limited fatalistic response after genetic risk feedback, measured by perceived control and self-reported fatalism^(^ ^51^ ^–^ ^53^ ^)^. Hence, this finding raises concern and warrants further testing and monitoring.

### Strengths and limitations of the review

This review strived to be comprehensive and included both published and unpublished literature, with efforts made to contact authors where necessary. Although the scope for publication bias may have been reduced considerably by direct correspondence with researchers and inclusion of previously unreported data, we recognise that residual publication bias deserves consideration and caution when interpreting results. However, there was an insufficient number of studies included to formally test for publication bias. None of the clinical studies examined potential mediators of behaviour change relevant to genetic testing, including genetic and health literacy and numeracy, and only two studies assessed baseline motivations^(^ ^21^ ^,^ ^29^ ^)^. Understanding how participants with differing characteristics respond to genetic information could assist in tailoring future delivery. The meta-analyses should be interpreted bearing in mind two limitations – one being that the heterogeneity in the data (i.e. measured outcome, condition and methods) restricted quantitative synthesis of all outcomes examined in the review, and two it was composed primarily of small studies based on the currently available literature. There was a lack of objectively measured behaviours, and the resulting measurement error of self-reported methods prevented firm conclusions to be drawn^(^ ^54^ ^)^. Hence, we look forward to the results from several ongoing studies that will provide the much-needed insight using objective methods^(^ ^22^ ^,^ ^55^ ^,^ ^56^ ^)^. Finally, none of the studies were from countries outside of the USA, Canada and Europe, which interestingly coincides with the availability of commercial personalised nutrition companies^(^ ^57^ ^)^. At present, an on-going study in Hong Kong will provide some much-needed perspectives from Asia^(^ ^58^ ^)^.

Further results from a meta-analysis on the effect of genetic risk on perceived control, effectiveness of intervention and risk can be found in the online Supplementary material 4.

### Conclusions, implications and recommendations for practice and research

On the basis of the totality of the evidence currently available for inclusion within this review, including both the meta-analyses and narrative synthesis, we found no clear or consistent evidence that genetic risk communication alone either raises motivation or translates into actual behaviour change to reduce the risk of cardiometabolic disorders in adults. With genetics proposed to influence health in multiple ways^(^ ^59^ ^)^, including genetic personalised nutrition^(^ ^1^ ^)^ accompanied by public enthusiasm, the incorporation of genetic risk into practice is likely to rise. Although we caution against unsupported online provision of genetic risk because of the lack of demonstrated clinical utility and possible negative implications, in the interim with absence of such evidence, dietitians/nutritionists may consider exploiting public enthusiasm in genetic risk as another opportunity to educate across a range of preventative lifestyle behaviours. This will require upskilling of the workforce in the area of genetics and genomics as we have previously demonstrated^(^ ^60^ ^,^ ^61^ ^)^.

Clearly, research is needed to untangle the effects that can be attributed to methods of personalisation from genetic risk communication for the impact on actual behaviour change. Specifically, larger-scale, high-quality clinical RCT with objective outcome measures are warranted. Participants should also be more thoroughly characterised to capture risk comprehension and initial level of motivation to enable health professionals to better tailor their risk feedback to these. Evaluation of clinical utility, alongside analytical and clinical validity, in addition to the ethical, legal and social implications of genetic testing, according to the Centre for Disease Control’s ACCE framework^(^ ^62^ ^)^, are current areas of enquiry. Finally, to ensure public welfare in engaging with genetic susceptibility information, policy makers need to enforce stricter regulation of DTC services, which could start by setting clear European frameworks.

## Supplementary material

For supplementary material accompanying this paper visit http://dx.doi.org/10.1017/S0007114516002488. click here to view supplementary material
