# Supplementary material for: The effect of communicating the genetic risk of cardiometabolic disorders on motivation and actual engagement in preventative lifestyle modification and clinical outcome: a systematic review and meta-analysis of randomised controlled trials
Source: Br J Nutr. 2016 Jul 13;116(5):924–34. doi: 10.1017/S0007114516002488 (PMC4983776; doi:10.1017/S0007114516002488)
Supplement: Supplementary file 1 [file S0007114516002488sup001.docx]

**Online Supplementary Materials**

**The effect of communicating the genetic risk of cardiometabolic disorders on motivation and actual engagement in preventative lifestyle modification and clinical outcome: a systematic review and meta-analysis of randomised controlled trials**

**Authors**

Sherly X. Li,^1^ Zheng Ye,^1^ Kevin Whelan,^2^ Helen Truby^3^

^1^Medical Research Council Epidemiology Unit, University of Cambridge, Cambridge, United Kingdom

^2^King's College London, Diabetes and Nutritional Sciences Division, London, United Kingdom

^3^Department of Nutrition & Dietetics, Monash University, Level 1, 264 Ferntree Gully Rd, Notting Hill, VIC, 3168, Australia

Corresponding author: Sherly X. Li

Medical Research Council Epidemiology Unit, University of Cambridge, Cambridge, United Kingdom

Email: [sherly.li@mrc-epid.cam.ac.uk](mailto:sherly.li@mrc-epid.cam.ac.uk)

**Supplementary Material 1: Sample of a search strategy log**

**EMBASE (date: 8-6-15)**

| **Searches** | **Search terms** | **Results** |
| --- | --- | --- |
| 1 | exp genetic services/ | 28762 |
| 2 | Exp genetic susceptibility/ | 26457 |
| 3 | (gene* NEXT/1 (test* OR assess* OR risk* OR susceptib* OR predispos* OR disease*)):de,ab,ti | 76612 |
| 4 | 'dna-based test':de,ab,ti OR (personali?ed NEXT/1 medicine):de,ab,ti OR (personali?ed NEXT/1 nutrition):de,ab,ti OR (nutritional NEXT/1 genomic*):de,ab,ti OR nutrigenetic*:de,ab,ti OR nutrigenomic*:de,ab,ti | 7342 |
| 5 | ‘direct-to-consumer genetic testing’ | 88 |
| 6 | (‘personal genome’ NEXT/1 test*): de,ab,ti | 16 |
| 7 | Or/1-6 | 103296 |
| 8 | Exp behavior change/ | 9116 |
| 9 | Exp health behavior/ | 106837 |
| 10 | Exp patient compliance/ | 51851 |
| 11 | adher*:de,ab,ti OR motivation:de,ab,ti OR interest:de,ab,ti OR motivation:de,ab,ti OR facilitate:de,ab,ti OR 'health decision':de,ab,ti OR 'risk reduction behavior':de,ab,ti | 239715 |
| 12 | Or/8-11 | 372870 |
| 13 | Exp obesity/ | 144254 |
| 14 | 'body weight':de,ab,ti OR 'body mass index':de,ab,ti OR bmi:de,ab,ti OR overweight:de,ab,ti | 186356 |
| 15 | weight NEXT/1 (gain OR loss OR change)):de,ab,ti | 64761 |
| 16 | Or/13-15 | 295520 |
| 17 | Exp non insulin dependent diabetes mellitus | 74389 |
| 18 | 'type 2 diabetes':de,ab,ti OR 'type two diabetes':de,ab,ti OR 'type 2 diabetes mellitus':de,ab,ti OR 'type two diabetes mellitus':de,ab,ti OR T2D:de,ab,ti OR niddm:de,ab,ti | 53995 |
| 19 | Or/17-18 | 82651 |
| 20 | Exp cardiovascular disease | 1022555 |
| 21 | cvd:de,ab,ti OR 'heart disease':de,ab,ti OR 'coronary artery disease':de,ab,ti OR hypercholesterol*:de,ab,ti OR hyperlipid*or:de,ab,ti AND lipoprotein:de,ab,ti OR atherosclerosis:de,ab,ti | 81517 |
| 22 | Or/20-21 | 1029403 |
| 23 | 22 or 19 or 16 | 1244747 |
| 24 | 23 and 12 and 7 | 2035 |
| **25** | **24 ([article]/lim OR [article in press]/lim OR [conference abstract]/lim OR [conference paper]/lim) AND [embase]/lim AND [2003-2014]/py** | **1034** |

**Grey literature search**

Sources: Proquest thesis (US), Trove (ANZ), ETHOS (UK), science.gov

Search terms: limited advanced searching function therefore selected search terms chosen

‘genetic test*’ AND ‘behavi* change’ AND ‘obes* OR diabetes OR cardiovascular disease’

Results: 0

**Reference mining**

Source: key review articles from database search and final studies for inclusion.

Results: 3 (4 papers. 1 study produced 2 publications)

**Key author search:**

Source: PubMed search of the following authors and ‘genetic’ as key word.

Ahmed El-sohemy (0), Michael Gibney (0), John Mathers (0), John Hesketh (0), Jane Wardle (0), Anna Macready (0), Barbara Stewart-Knox (0), Richard W Grant (1), Saskia C. Sanderson (0), Jason L Vassy (0), Cinnamon S Bloss (0), Theresa M. Marteau (0)

Results: 1 additional publication found

**WHO International clinical trials registry platform:**

Total:

Unpublished but completed trials: 5. Four authors contacted but did not respond.

Cho et al., NCT00849563

Godino et al., ISRCTN09650496

Egnatios et al., NCT01859403

Wang et al., NCT01355224

(limited results provided through personal correspondence)

Food4Me (White Paper available with limited results)

Incomplete: 2

1. INFORM study: ISRCTN17721237
2. Vorderstrasse et al., NCT01884545

**Supplementary Material 2: Summary table of Vignette studies**

| Study  (ref: author,yr) | Disease and other outcome measures | Gene | Participants, setting | Audience, medium | Comparison tests ( mean (SD) unless otherwise specified) | | | | | | |
| --- | --- | --- | --- | --- | --- | --- | --- | --- | --- | --- | --- |
| Outcome: Perceived motivation to change dietary behaviour | | | | | | | | | | | |
|  |  |  |  |  | **Control** | | **GT** | | **Alternative test** | | |
| Frosch et al., 2005  RCT (2x2F) | Obesity  Motivation to eat a healthy diet.  Attitudes about healthy eating.  Perceived control.  Outcome expectancies.  HR, AR | unspecified | n=249, USA  20.5 (1.7) yrs. | Scenario in survey  (1^st^ person). | **-** | | **GT**  NA | | **Hormone**  NA | | |
|  |  |  |  |  |  |  | No statistically significant difference | | | | |
| Sanderson et al., 2010  RCT (5P) | Obesity  Motivation to eat a healthy diet.  Perceived risk and severity of obesity.  Diet-response-efficacy.  Diet self-efficacy.  Diet and genetic causal beliefs.  Diet/metabolism. HR, AR. | Fictitious: *OB37* | n= 191, UK  29.2yrs | Scenario in survey  (1^st^ person). | **No risk** + generic advice | | **GT** + personalised advice | | **Enzyme** + personalised advice | | |
|  |  |  |  |  | No risk vs GT p=0.026, No risk vs enzyme p=0.002, GT vs enzyme: p=0.21. Overall: F(2,180)=5.12, p=0.007 | | | | | | |
| Smerecnik et al., 2009  RCT (2P)  Cholesterol | CVD: cholesterol  Motivation to reduce cholesterol.  Risk perception.  Awareness status. | Unspecified but predispose to higher cholesterol | n= 139, Netherlands  46yrs | General: population message.  Newspaper article with facts about cholesterol. | **General HM** | | **Genetic HM** | | - | | |
|  |  |  |  |  | HM type: β= -0.23, p=0.008  HM type x awareness interaction: β= -0.02, p=0.79 | | | | | | |
| Smerecnik et al., 2009  RCT (2P)  Salt sensitivity | CVD: hypertension  Motivation to reduce salt intake.  Risk perception.  Awareness status. | Unspecified but predispose to higher blood pressure | n= 293 (study 1A: 145, study 1B: 148)  Netherlands  Study 1A: 24yrs  Study 1B: 42yrs | General: population message.  Newspaper article with facts about hypertension. | **General HM** | | **Genetic HM** | | - | | |
|  |  |  |  |  | Unaware  Study 1A: p= 0.03, Study 1B: p<0.001  Aware  Study 1A: p= 0.17, Study 1B: p= 0.13  HM type: no effect  Study 1A: β= 0.49, p=0.26  Study 1B: β= 0.56, p=0.15  HM type x awareness interaction | | | | | | |
| Dar-Nimrod et al., 2015  RCT (3P) | Obesity (study3)  Eating behaviour: weight of cookies eaten (immediate outcome).  Food preferences and eating habits. | *GATA-2, FTO, KLF15* | n=162, Canada  20.8yrs | Newspaper article about causes of obesity (1^st^ person). | **Control**  No explanation given.  p = 0.08 | **Genetic** | | | | | **Psychosocial** |
|  |  |  |  |  |  | Those provided a genetic aetiology consumed more cookie  P=0.02 | | | | | |
|  |  |  |  |  |  | | | | | | |
| Outcome: Perceived control | | | | | | | | | | | |
| Frosch et al., 2005  RCT (2x2F) | Obesity  HR, AR | unspecified | n=249, USA  20.5 (1.7) yrs. | Scenario in survey (1^st^ person).  Likert scale -3 to +3 | **-** | | | **GT**  HR 1.4 (1.2)  AR 1.6 (1.0) | | **Hormone**  HR 1.6 (1.0)  AR 1.3 (1.2) | |
|  |  |  |  |  |  | | | **β 0.607, F statistic=77.89, p<0.001** | | | |
| Sanderson et al., 2010  RCT (5P) | Obesity  HR, AR. | Fictitious: *OB37* | n= 191, UK  29.2yrs | Scenario in survey (1^st^ person).  Likert scale +1 to +5 | **No risk information**  3.63 (0.83) | | | **GT**  3.68 (0.88) | | **Enzyme**  3.67 (0.84) | |
|  |  |  |  |  | F statistic= 0.43, p=0.65 | | | | | | |
| Outcome: Perceived effectiveness of treatment/intervention | | | | | | | | | | | |
| Frosch et al., 2005  RCT (2x2F) | Obesity  HR, AR | unspecified | n=249, USA  20.5 (1.7) yrs. | Scenario in survey (1^st^ person).  Likert scale -3 to +3 | **-** | | | **GT**  HR 1.77 (1.57)  AR 2.06 (1.27)  NA | | **Hormone**  HR 2.07 (1.18)  AR 1.9 (1.45) | |
| Sanderson et al., 2010  RCT (5P) | Obesity  HR, AR. | Fictitious: *OB37* | n= 191, UK  29.2yrs | Scenario in survey (1^st^ person).  Likert scale +1 to +5 | **No risk information**  4.07 (0.47) | | | **GT**  4.09 (0.63) | | **Enzyme**  4.11 (0.56) | |
|  |  |  |  |  | No statistically significant difference. F statistic= 0.28, p=0.74 | | | | | | |
| Outcome: Perceived risk | | | | | | | | | | | |
| Frosch et al., 2005  RCT (2x2F) | Obesity  HR, AR | unspecified | n=249, USA  20.5 (1.7) yrs. | Scenario in survey (1^st^ person).  Likert scale -3 to +3 | **-** | | | **GT**  HR: 3.23 (2.02)  AR: 2.89 (1.76) | | **Hormone**  HR: 3.79 (2.06)  AR: 3.07 (1.95) | |
|  |  |  |  |  |  | | | **β -0.746, F statistic=5.01, p<0.03** | | | |
| Sanderson et al., 2010  RCT (5P) | Obesity  HR, AR. | Fictitious: *OB37* | n= 191, UK  29.2yrs | Scenario in survey (1^st^ person).  Likert scale +1 to +5 | **No risk information**  2.22 (0.85) | | | **GT**  3.91 (0.84) | | **Enzyme**  3.55 (1.01) | |
|  |  |  |  |  | **F statistic=42.89, p<0.001** | | | | | | |

Abbreviations: GT: genetic testing; FH: family history; PA: physical activity; F: factorial; P: parallel; Audience 1^st^: first person; all: general; NE: no effect; HR: high risk; AR: average risk; LS: low severity; HS: higher severity; HM: health messages

**Supplementary Material 3: Summary table of clinical studies**

| **Study**  **(ref: author,yr)** | **Disease, outcome measures, genes analysed** | **Participants, setting, scale used, length of follow-up** | **Comparison tests** ( mean (SD) unless otherwise specified) | | | | | | | | | |
| --- | --- | --- | --- | --- | --- | --- | --- | --- | --- | --- | --- | --- |
| **Outcome: Actual motivations to change behaviour** | | | | | | | | | | | | |
| **Diet, PA** | | | **Control** | | **GT** | | | | | **Alternative test** | | |
| **Grant et al., 2013**  **RCT (3P)** | Type 2 diabetes  Confidence and motivation to make lifestyle changes.  Stage of change.  HR, AR | n= 108, USA  58.7yrs  3mth  n (%) of people with increased perceived risk | **Phenotypic risk + 12wk program** (n= 34)  Weight loss: 17 (50%)  Dietary change: 15 (44.1%)  Exercise: 11 (32.4%)  Diabetes prevention: 3 (8.8) | | **GT HR vs AR + genetic counselling + 12wk program** (HR: n=42, AR: n=32)  12 (28.6%) p=0.056, 13 (40.6%) p=0.44  16 (38.1%) p=0.60, 12 (37.5%) p=0.44  10(23.8%) p= 0.41, 8(25%) p=0.51  7 (16.7%) p=0.31, 5 (15.6%) p=0.4 | | | | | - | | |
|  | T2D genetic risk score (33 genetic variants) |  | No statistically significant difference | | | | | | |  | | |
| **Weight loss** | | |  | | | | | | |  | | |
| **Wang et al., unpublished (4P)** | Obesity  Motivation to change diet and exercise.  Attitudes and beliefs about obesity.  HR, AR | n=696, USA Multi-ethnic (93% White)  50yrs  6mths  60% overweight/obese  4 point Likert | **Control**  No risk feedback | **A**  Genetic risk feedback  HR vs AR | | | **B**  Lifestyle risk  HR vs AR | | | **C**  Lifestyle + genetic risk HR vs AR | | |
|  | Motivation to lose weight. |  | 3.06 (no sd) | 3.37 (no sd) | | | 3.27 (no sd) | | | 3.36 (no sd) | | |
|  |  |  | p<0.005 | | | |  | | |  | | |
|  | *FTO* |  | Stratification by BMI: higher motivation in obese/overweight (p=0.022) | | | | | | | | | |
| **Meisel et al., 2015 (2P)** | Obesity  Motivation to change diet and exercise.  Self-efficacy  HR, AR | n= 279, UK  21yrs, University students  BMI: 21kg/m^2^  1mth  5 point Likert | **Simple weight control advice** | | **GT based weight control advice** | | | | - | | | |
|  | Motivation for weight control |  | 1.5 (0.7) | | AR: 1.6 (0.8) vs HR: NA | | | |  | | | |
|  |  |  | Control vs AR  OR: 1.77 (95% CI = 1.08-2.89, p = 0.023) ↑  Control vs HR  OR: 2.38 (95%CI = 1.33-4.26, p = 0.003) ↑ | | | | | | - | | | |
|  | *FTO* |  | Stratification by BMI: higher motivation in obese/overweight (OR = 6.67, 95%CI 1.13-39.25, p= 0.036) | | | | | | | | | |
| **Outcome: Actual behaviour change** | | | | | | | | | | | | |
| **Grant et al., 2013**  **RCT (3P)** | Type 2 diabetes  Behavioural changes (class attendance for 12wk program).  HR, AR | n= 177, USA  56-61yrs  6mth | 6.6 (4.7) | | HR: 7 (4) (95%CI -1.6, 2.5, p=0.67) ↑  AR: 6.8 (4.2) (95%CI -1.9, 2.5, p=0.82) ↑  No statistically significant difference | | | | | | - | |
|  | Clinical change (weight loss- pounds). |  | 7.52 (9.59) | | HR: 8.74 (9.6) p=0.58 ↑  AR: 9.18 (11.6) p=0.53 ↑  No statistically significant difference | | | | | | - | |
|  | Clinical change (BMI change- kg/m^2^). |  | 1.02 (1.45) | | HR: 1.23 (1.47) p=0.52 ↑  AR: 1.3 (1.8) p=0.48 ↑  No statistically significant difference | | | | | | - | |
|  | Clinical change (lost 7% body weight- number of people). |  | 6 (17.7%) | | HR: 10 (23.8%) p=0.51 ↑  AR: 6 (18.8) p=0.91  No statistically significant difference | | | | | | - | |
|  | Diet and PA change |  | NA | | NA  No statistically significant difference | | | | | | - | |
| **Marteau et al., 2004 RCT (2P)** | CVD-Familial hypercholesterolemia  Risk reducing behaviour | n= 316, UK  54.9yrs  6mth | **clinical diagnosis + lifestyle advice** | | **clinical diagnosis + GT confirmation + lifestyle advice** | | | | | | - | |
|  | *LDAR, ApoB* |  | NA. No statistically significant difference | | | | | | | |  | |
| **Meisel et al., 2015 (2P)** | Obesity  Use of weight control strategies  HR, AR | n= 279, UK  21yrs, University students  BMI: 21kg/m^2^  1mth | **Simple weight control advice**  NA | | **GT based weight control advice**  NA. No statistically significant difference | | | | | | - | |
|  | Diet and PA change |  | NA | | NA | | | | | | - | |
|  | Weight change |  | 0.27 (3.08) | | HR: 1.53 (3.29)  AR: 1.23 (2.91) | | | | | | - | |
|  |  |  | OR = 0.73 (95%CI = 0.27-1.91, p = 0.523) No statistically significant difference | | | | | | | |  | |
| **Voils et al., 2015 (2P)** | T2D  Weight change | n= 601, USA multi-ethnic Veterans  54.1yrs  BMI≥27 kg/m^2^  3, 6mths | **conventional risk + control eye disease counselling** | | **conventional risk + genetic counselling** | | | | | | - | |
|  |  |  | 0-3mth: -0.4  3-6mth: 0 | | 0-3mth: -0.2  3-6mth: 0 | | | | | | - | |
|  |  |  | 3mth: 0.2 (95%CI -0.3-0.7, p=0.44) No statistically significant difference | | | | | | | | | |
|  | HOMA2-IR |  | 0-3mth: 0  3-6mth: 0.2 | | 0-3mth: 0.2  3-6mth: 0.2 | | | | | | - | |
|  |  |  | 3mth: 0.1 (95%CI -0.1-0.3, p=0.19) No statistically significant difference | | | | | | | | | |
|  | Diet intake (energy intake kcal/day) |  | 0: 1653  3mth: 1573  6mth: 1440 | | 0: 1653  3mth: 1487  6mth: 1312 | | | | | | - | |
|  |  |  | 3mth: -0.1 (95%CI:-0.2-0, **p=0.05** borderline)  6mth: -0.1 (95%CI:-0.1-0, p=0.20)  Other macronutrients: No statistically significant difference | | | | | | | | | |
|  | PA (walking intensity) |  | NA. No statistically significant difference | | | | | | | |  | |
|  | *TCF7L2, PPARγ, KCNJ1* |  |  | |  | | | | | |  | |
| **Hietaranta-Luoma et al, 2015 (2P)** | CVD risk  Diet intake (fat quality, F+V, fat and sugar, alcohol)  Leisure time PA  Health status and taste preference  HR: E4+, AR: E4- | n= 107, Finnish  47yrs  10weeks, 6 and 12 mths | **Health information**  (n=56) | | **Health information + genetic risk communication**  (E4+ n=16; E4- n=35) | | | | | |  | |
|  | Quality of fat intake | Scale 0-27 | 0: 16.3  10wk: 17.3 (0.5)  6mth: 17.6 (0.5)  12mth: 17.8 (0.5) | | 0: E4+: 16.3, E4-: 16.3  10wk: 20.1 (1.0), 18.1 (0.7)  6mth: 20.4 (0.9), 18.5 (0.6)  12mth: 18.7 (0.9), 18.3 (0.6) | | | | | | - | |
|  |  |  | Across groups: p<0.05**.** Significant difference also found for high fat and sugar containing foods. | | | | | | | |  | |
|  | PA (at least 2x30min/wk: %) |  | 0: 66.1 (37)  10wk: 69.6 (39)  6mth: 75.0 (42)  12mth: 64.3 (36) | | 0: E4+: 75.0 (12), E-: 80.0 (28)  10wk: 81.3 (13), 62.9 (22)  6mth: 75.0 (12), 74.3 (26)  12mth: 81.3 (13), 71.4 (25) | | | | | | - | |
|  | *ApoE* |  | NA. No statistically significant difference | | | | | | | |  | |
| **Food4Me, preliminary results, 2015 (4P)** | Obesity  BMI, waist circumference  Blood glucose and cholesterol | n=1607  European- multicentre  39.8yrs  BMI: 25.5kg/m^2^  3, 6mths | **Level 0: population health eating guidelines (no personalisation)** | | **Level 1: personalised dietary advice based on dietary intake assessment** | | | **Level2: level 1+ personalisation based on phenotypic and biomarkers** | | | **Level 3: level 2+ genetic data** | |
|  | Weight change |  | NA. No statistically significant difference in any of the weight groups including those overweight and/or obese. | | | | | | | | | |
|  | Dietary intake (FFQ)  HEI |  | NA (difference compared to control) | | 3mth:1.2  6mth: 1.15 | | | 3mth:1.2  6mth: 1 | | | 3mth:1.6  6mth: 1.9 | |
|  | *FTO, MTHFR, TCF7L2, ApoE, FADS1* |  | Results at 3mth  Level 0 vs L1-3: +1.4 (p<0.01) 6mth: +1.4 (p<0.05). A significant difference also reported for SFA and total fat intake.  Level 0 vs 1 vs 2 vs 3: No statistically significant difference | | | | | | | | | |
| **Outcome: Perceived control** | | | | | | | | | | | | |
| **Marteau et al., 2004 RCT (2P)** | CVD-Familial hypercholesterolaemia | n= 316, UK  44-56yrs  6mth  5 point Likert | **clinical diagnosis + lifestyle advice**  3.97 (0.90) | | **clinical diagnosis + GT confirmation + lifestyle advice**  mutation 4.01 (0.93)  no mutation 3.89 (1.00) | | | | | | | --- |
|  |  |  | mutation vs no mutation: (β 0.02, 95%CI -0.05 to 0.10, p=0.51)  mutation vs control: (β 0.01 95%CI -0.07 to 0.09, p=0.81)  No statistically significant difference | | | | | | | | |  |
| **Grant et al., 2013**  **RCT (3P)** | Type 2 diabetes  HR, AR | n= 108, USA  58.7yrs  3mths  n (%) of people with increased perceived confidence | **Phenotypic risk + 12wk program**  Weight loss: 18 (52.7)  Dietary change: 16 (47.1)  Exercise: 14 (41.2)  Diabetes prevention: 11 (32.4) | | **GT HR vs AR + genetic counselling + 12wk program**  15 (35.7), p=0.13, 18 (52.9), p=0.21  20 (47.6), p= 0.96, 11 (35.5), p=0.34  17 (40.5), p=0.95, 11 (34.4), p=0.57  17 (40.5), p=0.47, 13 (41.9), p=0.42 | | | | | | |  |
| **Outcome: Perceived effectiveness of treatment** | | | | | | | | | | | | |
| **Marteau et al., 2004 RCT (2P)** | CVD-Familial hypercholesterolaemia  Perceived treatment effectiveness: dietary (low fat diet) or medication | n= 316, UK  44-56yrs  6mth  7 point Likert | **clinical diagnosis + lifestyle advice**  Diet:  5.08 (1.29) | | **clinical diagnosis + GT confirmation + lifestyle advice**  mutation 4.79 (1.34)  no mutation 5.19 (1.09) | | | | | | - | |
|  |  |  | mutation vs no mutation: (β 0.40, 95%CI 0.05 to 0.75,p=0.02)  mutation vs control: (β 0.29, 95%CI -0.08 to 0.66,p=0.12) | | | | | | | |  | |
|  |  |  | Medication:  5.35 (0.93) | | mutation 5.54 (0.71)  no mutation 5.31 (0.86) | | | | | |  | |
|  |  |  | mutation vs no mutation: (β -0.24, 95%CI -0.48 to 0.01,p=0.06)  mutation vs control: (β -0.19, 95%CI -0.44 to 0.07,p=0.15) | | | | | | | |  | |
| **Outcome: Perceived risk** | | | | | | | | | | | | |
| **Grant et al., 2013**  **RCT (3P)** | Type 2 diabetes  HR, AR | n= 108, USA  58.7yrs  3mth  n (%) of people with increased perceived risk | **Phenotypic risk + 12wk program**  4 (11.8) | | | **GT HR vs AR + genetic counselling + 12wk program**  9 (22), p=0.25, 1 (3.1), p=0.18 | | | | |  | |
| **Voils et al., 2015 (2P)** | T2D  Weight change | n= 601, USA multi-ethnic Veterans  54.1yrs  BMI≥27 kg/m^2^  3, 6mth | **conventional risk + control eye disease counselling**  3mth: 3.1  6mth: 3.1 | | | **conventional risk + genetic counselling**  3mth: 3.0  6mth: 3.1 | | | | |  | |
|  |  |  | Treatment difference =0 (95%CI: -0.3 to 0.2), p=NA  No statistically significant difference | | | | | | | | | |

Abbreviations: GT: genetic testing; FH: family history; PA: physical activity; F: factorial; P: parallel; Audience 1^st^: first person; 3^rd^: third person; all: general; HR: high risk; AR: average risk; LS: low severity; HS: higher severity; HM: health messages; F+V: fruit and vegetable; PA: physical activity; T2D: type 2 diabetes; NA: not available

**Supplementary Material 4: Meta-analyses of the effect of genetic risk communication on psychological outcomes**

The following are potential mediators that have been previously reported in the literature, which were examined in some vignette and some clinical studies. Below we present the summarised findings in text. We also conducted meta-analyses of the vignette studies because of the greater number of studies available. These were not combined with results from clinical studies because of the differences in study design (see Figure 4).

*Perceived control*

Two clinical^[1,2]^ and two vignette studies^[3,4]^ revealed that perceived control for behaviour change (diet, exercise or diabetes prevention) was unaffected by the provision of genetic risk information. Even after being diagnosed with a monogenic condition such as FH, control over FH, cholesterol levels and CVD risk were similar between intervention groups.^[2]^ Frosch et al., however detected lower perceived control in those presenting with a higher genetic risk than those with average genetic risk. Contrasting results were reported for the hormone risk estimate.^[4]^ Our meta-analysis does not support the notion that genetic risk information influences perceived control (SMD: -0.09, 95% CI: -0.48 to0.81, p=0.66), in the context of two small RCTs.

*Perceived effectiveness of intervention*

Three studies^[2–4]^ examined how effective an intervention was after genetic risk information was provided to participants. Compared with controls, there were no differences in perceived efficacy of behavioural versus medical treatments for FH, cholesterol control or CVD prevention.^[2]^ When stratified by level of genetic risk, diet was considered more effective among those with average genetic risk whilst medication was more effective for those with high genetic risk.^[2]^ Pooled results from the meta-analysis found no association between the type of risk information (genetic versus alternative) on perceived effectiveness of either intervention (SMD: -0.13, 95%CI: -0.52 to 0.26, p=0.52).

*Perceived risk*

Five studies explored if genetic risk information may impact on perceived risk of developing the metabolic condition of interest (two clinical^[5,1]^ and three vignette studies).^[3,4,6]^ Pooled results from vignette studies were consistent with clinical studies, showing no difference between genetic test and control (based on a standardised likert scale 1- 10, random effects meta-analysis difference of: 0.52, 95%CI: -0.66 to 1.7, p=0.386) or between genetic and alternative test (0.021, 95%CI: -1.464 to 1.506, p=0.978). High heterogeneity appears to be due to differences in the method of communicating risk. Sanderson et al., communicated genetic risk using the first person,^[3]^ which may be more salient than the third person approach adopted by Smerecnik et al.,.^[6]^ This may explain the heterogeneity in the meta-analysis on the perceived risk of disease (genetic vs control). Whereas the high heterogeneity observed in the meta-analysis between communicating risk using a genetic vs an alternative test may be because Frosch et al.,^[4]^ appeared to possess higher risk of bias than Sanderson et al.,^[3]^ owing to lack of information on allocation concealment and selective reporting of results.

**Supplementary figure 4: Summary of pooled standardised mean difference (SMD) in perceived confidence, effectiveness of behaviour change and risk of disease, via a random effects meta-analysis of vignette studies (standardised likert scale: 1 to 10)**. I^2^ is the between-trial heterogeneity.


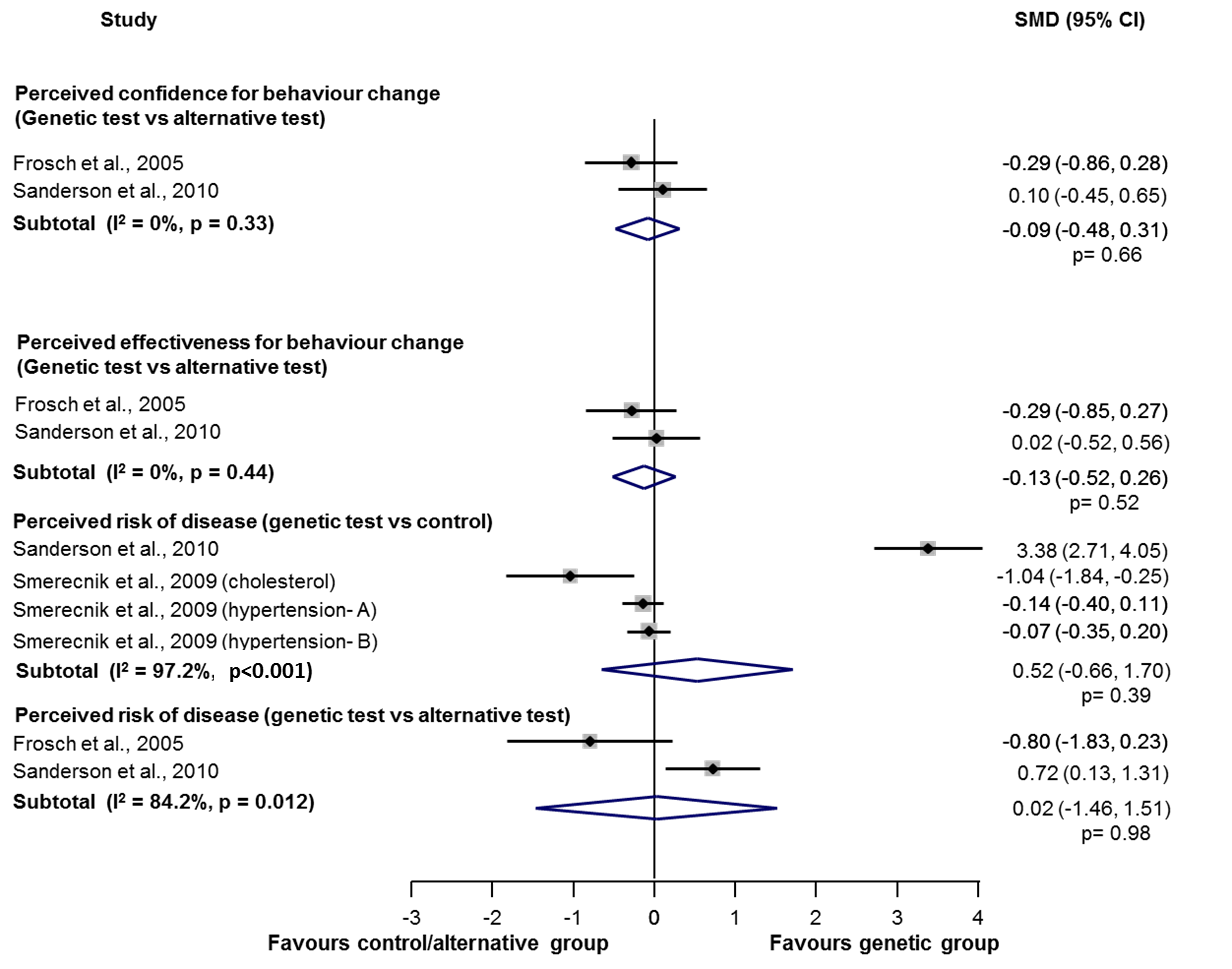


**References**

1. Grant RW, O’Brien KE, Waxler JL, et al. Personalized Genetic Risk Counseling to Motivate Diabetes Prevention. A RCT. Diabetes Care 2013;36:13–9.

2. Marteau T, Senior V, Humphries SE, Bobrow M, Cranston T, Crook M a, et al. Psychological impact of genetic testing for familial hypercholesterolemia within a previously aware population: a randomized controlled trial. Am J Med Genet 2004;128A:285–93.

3. Sanderson SC, Persky S, Michie S. Psychological and behavioral responses to genetic test results indicating increased risk of obesity: does the causal pathway from gene to obesity matter? Public Health Genomics 2010;13(1):34–47.

4. Frosch DL, Mello P, Lerman C. Behavioral Consequences of Testing for Obesity Risk. Cancer Epidemiol biomarkers Prev 2005;14(6):1485–9.

5. Voils CI, Coffman CJ, Grubber JM, Edelman D, Sadeghpour A, Maciejewski ML, et al. Does Type 2 Diabetes Genetic Testing and Counseling Reduce Modifiable Risk Factors? A Randomized Controlled Trial of Veterans. J Gen Intern Med 2015;30(11):1591–8.

6. Smerecnik CMR, Mesters I, de Vries NK, de Vries H. Alerting the general population to genetic risks: the value of health messages communicating the existence of genetic risk factors for public health promotion. Heal Psychol 2009;28(6):734–45.
